# Preschoolers show less trust in physically disabled or obese informants

**DOI:** 10.3389/fpsyg.2014.01524

**Published:** 2015-01-06

**Authors:** Sara Jaffer, Lili Ma

**Affiliations:** Department of Psychology, Ryerson UniversityToronto, ON, Canada

**Keywords:** trust, testimony, informant characteristics, physically disabled or obese, past reliability

## Abstract

This research examined whether preschool-aged children show less trust in physically disabled or obese informants. In Study 1, when learning about novel physical activities and facts, 4- and 5-year-olds preferred to endorse the testimony of a physically abled, non-obese informant rather than a physically disabled or obese one. In Study 2, after seeing that the physically disabled or obese informant was previously reliable whereas the physically abled, non-obese one was unreliable, 4- and 5-year-olds did not show a significant preference for either informant. We conclude that in line with the literature on children’s negative stereotypes of physically disabled or obese others, preschoolers are biased against these individuals as potential sources of new knowledge. This bias is robust in that past reliability might undermine its effect on children, but cannot reverse it.

## INTRODUCTION

The physical characteristics of an individual are a major source of influence on how we form impressions of and interact with other people (e.g., Feingold, 1992; Zebrowitz and Franklin, 2014). In particular, physical differences that are visually salient (e.g., physically disabled or obese) may give rise to social biases across a range of dimensions (e.g., Yuker, 1994; Puhl and Heuer, 2009). Research has demonstrated that such biases emerge early in development. By the preschool years, children have already developed negative stereotypes of physically disabled or obese others, which may foster prejudice toward and discrimination against these individuals in social interactions (e.g., Richardson, 1983; Favazza and Odom, 1997; Cramer and Steinwert, 1998; Wei and Di Santo, 2012). The present research aims to explore whether preschoolers display similar biases in contexts other than social judgments and interactions, such as when learning new knowledge from the testimony of others.

Children acquire a great deal of knowledge from the people around them (Harris and Koenig, 2006; Harris, 2007). It has been argued that young children may have a natural tendency to trust what they are told by others (Dawkins, 1995). Supporting this, 3-year-olds are credulous toward false claims of an adult that contradict their own firsthand observations (Ma and Ganea, 2010). Nevertheless, studies have shown that when the claims of two informants are placed in direct contrast with each other, children as young as 3 take into account various informant characteristics to guide their selective learning. For example, when learning about novel object labels or functions, preschoolers prefer to endorse the testimony of an informant who is reliable in the past rather than a previously unreliable one (e.g., Koenig et al., 2004; Einav and Robinson, 2010; Liu et al., 2013). Children also attend to the social markers of informants. It has been shown that preschoolers prefer to learn about novel objects from adults rather than children (e.g., Jaswal and Neely, 2006), from speakers of their own gender rather than the opposite gender (Ma and Woolley, 2013, Study 1), and from native-accented rather than foreign-accented speakers (e.g., Kinzler et al., 2011). Among these cues, past reliability seems a stronger indicator of informant trustworthiness for children than other cues such as age (Jaswal and Neely, 2006) and accent (Corriveau et al., 2013).

In addition to past reliability and social group membership, recent findings suggest that young children also use the physical characteristics of informants to guide their selective learning. For example, when presented with a physically stronger puppet versus a weaker one, 4- to 5-year-olds are more likely to endorse a novel object label provided by the stronger puppet (Fusaro et al., 2011). Also, 4- and 6-year-olds perceive a professionally dressed individual to be more knowledgeable than a casually dressed one, and prefer to learn novel labels from the former (McDonald and Ma, under review). In addition, when two informants, one with a more attractive and one with a less attractive face, provide conflicting labels for a novel object, 4- and 5-year-olds prefer to endorse the testimony of the informant with a more attractive face (Bascandziev and Harris, 2014). Despite these interesting findings, no studies have specifically examined if an informant’s physical capability or body type, such as being physically disabled or obese, would have an impact on children’s selective learning from others. The present study aims to address this question.

Past research has demonstrated that by the preschool years, children have developed biases against unfamiliar individuals with a physical disability (Favazza and Odom, 1997; Roberts and Smith, 1999). For example, studies have shown that preschoolers prefer a physically abled adult or peer rather than a disabled one for joint activities requiring high levels of physical involvement (Popp et al., 1981; Cohen et al., 1994). In addition, both physically abled and handicapped children prefer (from most to least) a non-disabled peer, a peer with crutches or brace, a peer in a wheelchair, a peer without a hand, a peer with a facial abnormality, and an obese peer (Richardson et al., 1961; Richardson, 1983). Moreover, preschoolers are biased to view physically disabled others as less competent than physically abled counterparts (Diamond and Hestenes, 1996). However, some research shows that negative attitudes toward physically disabled others do not persist over the childhood years, and that children may develop more positive attitudes toward these individuals in late childhood (Spillers, 1982). Nevertheless, it has been shown that between the ages of 7 and 11 years, children’s positive attitudes toward the physically disabled may decline and become negative when considering personal involvement in activities with these individuals (Magiati et al., 2002).

Biases against unfamiliar individuals with obesity also emerge by the preschool years, and become increasingly stronger in late childhood (Latner and Stunkard, 2003; Tillman et al., 2007). Since there is a great importance placed on physical appearance from an early age, overweight or obese children are at great risk for being stigmatized (Weil, 1977). Indeed, many studies have shown that children as young as 3 hold negative attitudes toward obese peers, and that preschoolers attribute more negative traits to obese than average-weight peers (e.g., Cramer and Steinwert, 1998; Musher-Eizenman et al., 2004; Wei and Di Santo, 2012). For example, 3-year-olds view overweight peers as mean, lazy, unattractive, unhappy, unpopular, unfriendly, and less intelligent (Wei and Di Santo, 2012). Even obese children themselves have negative stereotypes of other obese individuals (Staffieri, 1967; Lerner and Korn, 1972). In addition, preschoolers are biased against obese peers in playmate selection, and prefer to interact with average-weight rather than obese peers (Bell and Morgan, 2000; Musher-Eizenman et al., 2004).

Together, these research findings indicate that preschool-aged children have already developed negative stereotypes of physically disabled or obese individuals that may foster prejudice and discrimination. Such biases may be far-reaching and influence young children’s learning from unfamiliar people. For example, preschoolers might show less trust in physically disabled or obese individuals as potential sources of new knowledge, especially when their testimony is in direct contrast with that of physically abled, non-obese informants. This potential bias against physically disabled or obese informants might vary depending on whether or not there are available cues for the informants’ past reliability. We examined these questions in two studies.

## STUDY 1

The goal of Study 1 was to examine whether 4- and 5-year-olds would selectively endorse the testimony of a physically abled, non-obese informant versus a physically disabled or obese one. The two informants provided conflicting statements about novel physical activities (four trials) or novel facts (four trials), and children were asked to endorse the testimony of only one informant. The novel physical activities required high levels of physical involvement and thus were dependent on one’s physical capability or body type. In contrast, the novel facts were unrelated to one’s physical conditions and considered domain general. We focused our investigation on 4- and 5-year-olds for two reasons. As reviewed earlier, this age range has been the most frequently used in the literature on children’s selective trust in others, and social biases against physically disabled or obese individuals are already in place during the preschool years.

It was hypothesized that overall children would prefer the testimony of the physically abled, non-obese informant rather than the disabled or obese one. In addition, it was expected that this preference would be stronger in the domain of physical activities based on the principle of familiarity: compared to physically abled, non-obese individuals, physically disabled or obese people might be less likely to engage in the novel physical activities and thus less knowledgeable about this domain.

### METHOD

#### Participants

Participants were 47 children, including 23 4-year-olds (*M* = 52.3 months, range = 48.4–56.0 months; 11 girls) and 24 5-year-olds (*M* = 64.0 months, range = 60.2–69.7 months; 12 girls). Most participants were White (29), with some Asian (6), some African American (3), some Other Race (5), and some Mixed Race (4) participants. Two additional children were tested but excluded from the final sample because of language barriers or lack of attention. All children were recruited from the Greater Toronto Area through a participant database, various childcare programs, and the Ontario Science Centre. This research was approved by the Research Ethics Board at Ryerson University in Toronto, Canada.

#### Materials and stimuli

***Materials*.** Photos of eight adults (four women and four men) were used during the study, two photos per person. Each person appeared physically abled and non-obese in one photo, and physically disabled (a man in a wheelchair or a woman missing both arms) or obese in the other photo that was edited through Photoshop. Four audio clips were obtained from each person. Two clips contained correct versus incorrect statements about a novel physical-activity, and the other two contained correct versus incorrect statements about a novel fact (32 clips in total). Two content images were used for each of the eight test trials, one depicting the correct statement and the other one depicting the incorrect statement (16 content images in total). In addition, one topic image was used for each test trial, which depicted an object (or a group of objects) associated with the learning subject of the test trial (eight topic images in total).

The parent/guardian completed a demographics questionnaire about the child, which included general questions such as the child’s age and ethnicity, as well as more specific questions regarding if the child had been exposed to physically disabled or obese individuals (e.g., whether or not the child knows a person with disabilities or obesity; if yes, who the person is and how often the child interacts with that person).

**FIGURE 1:**
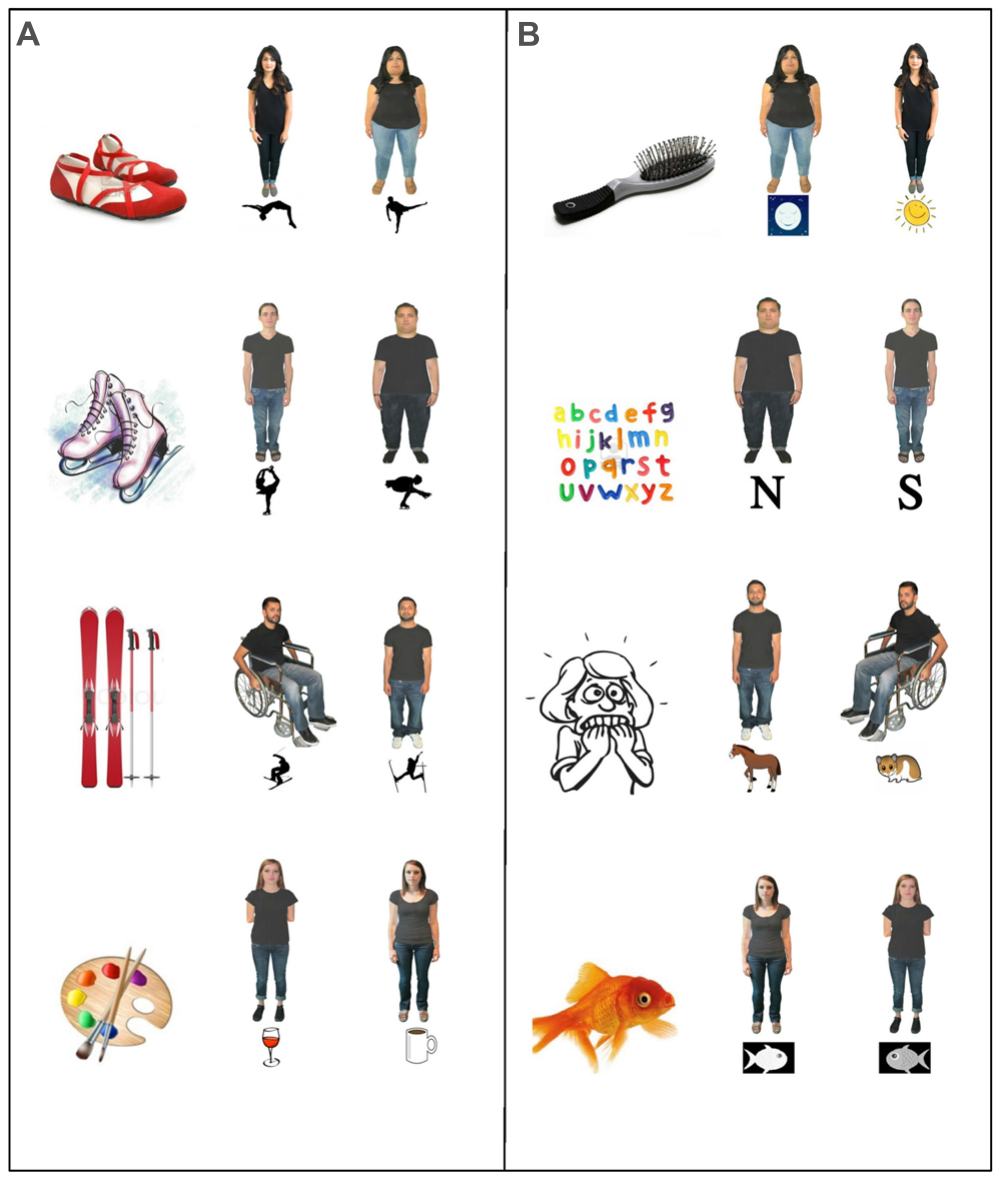
**Sample stimuli in Study 1.** PowerPoint presentations for **(A)** four physical-activity trials (from the top: a salto move in gymnastics; the Biellmann spin in skating; the Daffy move in skiing; the Arfé technique in painting), and **(B)** four fact trials (from the top: at what time of the day a human’s hair grows the fastest, in the morning or in the evening; which letter in the alphabet is used less, S or N; hippophobia is the fear of which animal, horses or hamsters; what color a goldfish changes to when it is kept in a dark room, white or gray). Two slides were used for each trial, with the topic image on the first slide (left) and the two informants and the content images on the second slide (right).

***Stimuli*.** The test stimuli consisted of 18 PowerPoint slides on a laptop with speakers. Apart from an introduction slide and a closing slide, each of the eight test trials consisted of two slides: the first slide with a topic image, and the second slide with photos of two adults with their audio clips embedded and two content images (one underneath each photo). A total of four pairings of informants were used on the four physical-activity trials and then again on the four fact trials, including two pairings for the disabled trials (a physically abled man vs. a man in a wheelchair, a physically abled woman vs. a woman missing both arms) and two for the obese trials (a non-obese man vs. an obese man, a non-obese woman vs. an obese woman). Each pair of informants was matched in terms of age, race, skin color, hair color, and outfits. A team of adults reviewed each pair of informants as equally confident in their claims and equally attractive before the photo editing took place. On each of the physical-activity trials, the two informants provided conflicting definitions of a novel physical-activity. On each of the fact trials, the two informants provided conflicting statements about a novel fact. A team of adults discussed the plausibility of different facts. Their feedback and suggestions were incorporated into choosing the best pairing of facts for each trial. The pairings were further refined based on the feedback and suggestions from the parents and children of the pilot testing. See **Figure 1** for sample stimuli.

Each child was randomly assigned to receive the test trials in one of two predetermined, randomized orders. The sides of the two informants and who provided the correct answer on each trial were counterbalanced across participants. The physical characteristic of each informant was also counterbalanced: each informant appeared physically abled and non-obese for half of the participants, and physically disabled or obese for the other half of the participants.

#### Design and procedure

The study employed a 2 (domain: activity vs. fact) × 2 (informant characteristic: disabled vs. obese) × 2 (age) mixed design, with domain and informant characteristic as the within-subjects factors, and age as the between-subjects factor. Each child completed four physical-activity trials and four fact trials, with two disabled trials and two obese trials within each domain. The study took place in a quiet room where the child was seated on a chair in front of a laptop. A video camera recorded the child’s responses throughout the study. The procedure included three phases: introduction, test, and interview.

***Introduction phase*.** After a warm-up session, a female researcher directed the child to the testing room. Pointing to the introduction PowerPoint slide on the laptop computer, the researcher told the child, “Today we’re going to learn about some new things from different people. Let’s begin!” The test phase followed.

***Test phase*.** On each of the eight trials, the researcher first showed the slide with the topic image, and asked the child to label the object presented (e.g., a pair of skis). Once the child correctly identified the object, the researcher asked the child whether he or she knew how to do a novel physical-activity or knew about a novel fact while pointing to the topic image (e.g., “Do you know how to do the skiing move called the Daffy?” or “At what time of the day does a human’s hair grow the fastest, in the morning or in the evening?”). None of the children knew about the novel physical activities or facts used in the study. Next, the researcher introduced the second slide depicting two adult informants and told the child, “One of them knows better. Let’s listen to what they say!” Then the audio clip of each informant’s testimony was played for the child (left side first) and was repeated by the researcher. Afterward, the child was asked to indicate how he or she would perform the physical-activity or what was the correct answer to the novel fact, by choosing to endorse the testimony of only one of the informants. After the child provided a clear response either verbally or by pointing, the procedure was repeated for the next trial.

***Interview phase*.** After the test phase, the researcher asked the child to justify his or her choices on two physical-activity trials and two fact trials, “Why did you choose him/her instead of him/her (pointing)?” If the child would not answer, a prompt was provided (i.e., “Why did you think he/she knew better about____?”).

At the end of the study, the child was thanked for his or her participation and received a debriefing of what the novel physical activities were and the correct answers to the novel facts.

#### Coding and reliability

The researcher (i.e., the initial coder) coded children’s responses during the testing session. A trained undergraduate research assistant performed reliability coding on a randomly selected 50% of the sample, and achieved 100% agreement with the initial coder.

### RESULTS

#### Children’s testimony endorsement

Preliminary analyses indicated that within each domain (activity vs. fact), children responded similarly on the disabled and the obese trials, so informant characteristic was not included in the main analyses here. All reported *p* values are two-tailed.

For each child, we calculated the proportion of the physical-activity trials and the proportion of the fact trials on which the child endorsed the testimony of the physically abled, non-obese informant. **Table 1** shows the means and SDs.

**Table 1:** Mean proportion of trials (out of four) choosing the physically abled, non-obese informant, by domain and age (SD in parentheses).

Age	*N*	Domain		Total
		Activity	Fact	
4-year-olds	23	0.65 (0.223)	0.58 (0.296)	0.61 (0.164)
5-year-olds	24	0.57 (0.299)	0.70 (0.208)	0.64 (0.180)
Total	47	0.61 (0.265)	0.64 (0.260)	0.63 (0.171)

Since the dependent variable was multinomial, we conducted a repeated-measures generalized linear model with generalized estimating equations (GEE), with domain as the within-subjects factor and age as the between-subjects factor. The results showed that the main effect of domain was not significant, Wald χ^2^ = 0.11, *p* = 0.74; nor was the main effect of age, Wald χ^2^ = 0.34, *p* = 0.56. No significant interaction emerged, Wald χ^2^ = 2.12, *p* = 0.15.

We then compared children’s choices to chance expectation (0.50), collapsing data across the two age groups. One-sample *t*-tests indicated that on average, children chose the physically abled, non-obese informant significantly more often than would be expected by chance, on both the physical-activity and the fact trials, *t*(46) = 2.89, *p* = 0.006, Cohen’s *d* = 0.42, and *t*(46) = 3.65, *p* = 0.001, Cohen’s *d* = 0.54, respectively.

#### The role of children’s prior exposure

Further analyses were conducted to examine the role of children’s prior exposure to physically disabled or obese individuals. Children whose parents were unsure of their exposure or did not respond to the questions were excluded from the analyses. Children’s choices were examined separately by informant characteristic, in relation to their prior exposure to individuals who were physically disabled (for the disabled trials, *n* = 36) or obese (for the obese trials, *n* = 36). **Table 2** shows the means and SDs.

**Table 2:** Mean proportion of trials (out of four) choosing the physically abled, non-obese informant, by trial type and prior exposure (SD in parentheses).

Trial Type	Exposure	*N*	Mean
Disabled	Yes	11	0.59 (0.280)
	No	25	0.60 (0.204)
Obese	Yes	21	0.60 (0.321)
	No	15	0.72 (0.186)

Independent-samples *t*-tests indicated that the children with or without relevant exposure responded similarly [disabled trials: *t*(34) = 0.11, *p*= 0.91; obese trials: *t*(34) = -1.31, *p* = 0.20]. However, the children without relevant exposure chose the physically abled, non-obese informant significantly above chance [disabled trials: *t*(24) = 2.45, *p*= 0.022, Cohen’s *d* = 0.49; obese trials: *t*(14) = 4.52, *p*< 0.001, Cohen’s *d* = 1.14]. Interestingly, the performance of the children with relevant exposure did not differ significantly from chance [disabled trials: *t*(10) = 1.08, *p*= 0.31; obese trials: *t*(20) = 1.36, *p*= 0.19].

#### Children’s justifications for their choices

Each of the 47 children was asked to justify his or her choices on four of the test trials, with a potential total of 188 responses. The researcher forgot to ask the interview questions on 10 occasions. Thus, a total of 178 responses were obtained from children, which were coded into the following five categories: (a) referencing the physical characteristic of the chosen informant (e.g., “because she’s bigger”; 1/178, <1%), (b) no explicit justification (e.g., “I just guessed” and “I don’t really know”; 58/178, 33%), (c) superficial judgments of the chosen informants (e.g., “because he is right” and “because she said so”; 24/178; 13%), (d) referencing the testimony itself (e.g., “because you skate on one leg and put your other leg up”; 55/178, 31%), and (e) Responses unrelated to the testimony (e.g., “lights! She’s in the street”; 40/178, 22%).

### DISCUSSION

The results of this study suggested that a speaker’s physical capability or body type did have an impact on children’s selective learning from others. When learning about novel physical activities and facts, 4- and 5-year-olds preferred the testimony of the physically abled, non-obese informants to that of the physically disabled or obese ones, across both domains (physical activities and facts) and both informant characteristics (disabled and obese). This bias against the physically disabled or obese informants is consistent with previous findings that by the preschool years, children have already developed negative stereotypes of physically disabled or obese individuals that may lead to prejudice and discrimination (e.g., Richardson, 1983; Favazza and Odom, 1997; Cramer and Steinwert, 1998; Wei and Di Santo, 2012).

When looking at the influence of prior exposure to disabled or obese individuals on children’s performance, the results showed that the children with and without relevant exposure responded similarly. However, the children without relevant exposure chose the physically abled, non-obese individuals significantly above chance on both the disabled and the obese trials. The performance of the children with previous exposure did not differ significantly from chance. We will return to this finding in General Discussion.

Children’s responses to the interview questions suggest that they did not explicitly consider the physical characteristics of the informants when making their choices (with the possible exception of one child). Their explanations were mostly superficial and unsophisticated in nature. One possibility is that at the age of 4 or 5, due to relatively limited language and/or cognitive resources, children were not able to explicitly articulate their reasons for choosing one informant over the other, although they might have attended to the physical characteristics of the informants at an implicit level. Another possibility is that children were showing sophistication in their responses by not pointing out the informant’s disability or obesity, and were trying to be politically correct and polite. Supporting this, Talwar and Lee (2002) found that children as young as 3 engaged in “white lie telling,” as they told an experimenter with a red mark on her nose that she looked fine just before her picture was taken. However, once the experimenter had left the room, the children expressed that she did not in fact look okay due to the mark on her nose.

To summarize, Study 1 showed that 4- and 5-year-olds displayed an overall preference to learn novel information from the physically abled, non-obese informants. In other words, they chose not to learn from the physically disabled or obese informants. How robust is this bias against potential informants with a physical disability or obesity? We conducted Study 2 to examine this question.

## STUDY 2

Studies have shown that past reliability trumps other cues such as age and accent in guiding children’s selective trust in testimony (Jaswal and Neely, 2006; Corriveau et al., 2013). Moreover, young children’s initial credulity toward the false testimony of an adult can be reversed by a single exposure to the adult as previously unreliable (Ma and Ganea, 2010). In light of these findings, in Study 2 we pitted physical characteristic against past reliability. Four- and 5-year-olds were shown that the physically disabled or obese informant was previously reliable whereas the physically abled, non-obese one was previously unreliable. It was hypothesized that such evidence would undermine children’s bias against the physically disabled or obese informant. Because the children in Study 1 responded similarly across domains, in Study 2 participants received only the four physical-activity trials. Also, the interview phase was omitted due to the lack of informative responses from children as discussed earlier.

### METHOD

#### Participants

Participants were 47 children, 24 4-year-olds (*M* = 53.5 months, range = 48.1–59.1 months; 12 girls) and 23 5-year-olds (*M* = 65.7 months, range = 60.4–71.2 months; 10 girls). Most participants were White (24), with some Asian (13), some African American (2), some Other Race (5), and some unidentified participants (3). Five additional children were tested but excluded from the final sample because they were out of the age range (2), did not complete the procedure (2), or did not pass the history trials (1).

#### Materials and stimuli

The materials for the four physical-activity trials in Study 1 were used, with the addition of the following: images of four familiar objects (ball, blocks, apple, butterfly), and two audio clips from each of the eight adults labeling one of the familiar objects correctly or incorrectly. The stimuli consisted of 14 PowerPoint slides: the introduction slide, 12 slides for the four test trials, and the closing slide. Each test trial had three slides: the history slide on which the two informants provided conflicting labels for the familiar object, followed by the same two slides used on each physical-activity trial as in Study 1. On each history slide, the physically disabled or obese informant was always correct in labeling the familiar object whereas the physically abled, non-obese informant was always incorrect. The counterbalancing was done in the same way as in Study 1.

#### Design and procedure

The study employed a mixed design, with informant characteristic (2: disabled vs. obese) as the within-subjects factor and age (2) as the between-subjects factor. The procedure included an introduction phrase and a test phase. After the child was introduced to the task as in Study 1, the test phase with four trials began. Each trial consisted of two sessions: history and endorsement. In the *history session*, the physically disabled or obese informant labeled a familiar object correctly whereas the physically abled, non-obese informant labeled it incorrectly. This was to show children that the physically abled and non-obese informant was unreliable as a source of information. First, the child was asked to label the familiar object (“Do you know what this is?”). All children correctly labeled all four familiar objects. Next, the researcher pointed to the two informants and told the child, “Let’s listen to what they call it. One of them knows better.” Then, the researcher played the audio clip of each informant labeling the familiar object for the child, and repeated each informant’s testimony. Afterward, the child was asked to identify which informant was correct (all children correctly responded to this question on each trial). The *endorsement session* followed, which was identical to the corresponding session in Study 1.

#### Coding and reliability

As in Study 1, the researcher coded children’s responses during the study, and an undergraduate research assistant coded a randomly selected 50% of the sample for reliability. There was no disagreement between the two coders.

### RESULTS AND DISCUSSION

For each child, we calculated the proportion of the disabled trials and the proportion of the obese trials on which the child endorsed the testimony of the physically abled, non-obese informant. A repeated-measures generalized linear model with GEE revealed that there was no significant main effect of informant characteristic or age, Wald χ^2^ = 0.63, *p* = 0.43, and Wald χ^2^ = 2.16, *p* = 0.14, respectively. No significant interaction was found, Wald χ^2^ = 0.14, *p* = 0.71.

Children’s choices did not differ significantly from chance expectation (0.50), on both the disabled (*M* = 0.49, SD = 0.423) and the obese trials (*M* = 0.41, SD = 0.421), *t*(46) = -0.17, *p* = 0.86, and *t*(46) = -1.39, *p* = 0.17, respectively.

We further examined children’s testimony endorsement in relation to their prior exposure to physically disabled or obese individuals, with valid data from 38 children for the disabled trials and 36 for the obese trials. **Table 3** shows the means and SDs. Independent-samples *t*-tests indicated that the children with or without relevant exposure responded similarly [disabled trials: *t*(36) = 0.85, *p* = 0.40; obese trials: *t*(34) = 0.26, *p* = 0.80]. In addition, children’s performance (with or without exposure) did not differ significantly from chance expectation [disabled trials: *t* (14) = 0.29, *p*= 0.77, and *t*(22) = -1.00, *p*= 0.33, respectively; obese trials: *t*(15) = -0.57, *p*= 0.58, and *t*(19) = -1.07, *p*= 0.30, respectively].

**Table 3:** Mean proportion of trials (out of two) choosing the physically abled, non-obese informant, by trial type and prior exposure (SD in parentheses).

Trial Type	Exposure	*N*	Mean
Disabled	Yes	15	0.53 (0.442)
	No	23	0.41 (0.417)
Obese	Yes	16	0.44 (0.443)
	No	20	0.40 (0.417)


For exploratory purposes, we compared children’s overall performance to that of the children in Study 1 (on the physical-activity trials only). An independent-samples *t*-test indicated that children were less likely to choose the physically abled, non-obese informant who was previously unreliable (Study 2: *M* = 0.45, SD = 0.264) than when there was no evidence of previous reliability (Study 1: *M* = 0.61, SD = 0.265), *t*(92) = -2.93, *p*= 0.004, Cohen’s *d* = 0.60.

In summary, Study 2 showed that when physical characteristic was pitted against past reliability, children did not display a statistically significant preference for either informant. Our informal comparison across studies suggested that past reliability might undermine children’s bias against the physically disabled or obese informant to some degree.

## GENERAL DISCUSSION

The two studies reported here examined whether preschoolers showed less trust in the testimony of physically disabled or obese informants. The main results indicated that when learning about novel physical activities and facts, 4- and 5-year-olds had an overall preference for the physically abled, non-obese informants over the physically disabled or obese ones. In other words, they displayed a bias against the physically disabled or obese informants (Study 1). When the physically disabled or obese informant was previously reliable whereas the physically abled, non-obese one was previously unreliable, 4- and 5-year-olds did not show a significant preference for either informant (Study 2). Each of these main findings is discussed in more detail below.

In Study 1, 4- and 5-year-olds preferred to endorse the testimony of the physically abled, non-obese informants. Their bias against the physically disabled or obese informants is in line with previous literature indicating that from an early age, children display prejudice toward unfamiliar individuals with physical disabilities or obesity and attribute various negative traits to them (e.g., Favazza and Odom, 1997; Cramer and Steinwert, 1998; Wei and Di Santo, 2012). Furthermore, children showed this bias across domains of both physical activities and facts. Based on the principle of familiarity or the cognitive division of labor, it was expected that children would perceive the physically disabled or obese informants to be less familiar with and thus less knowledgeable about novel physical activities because of their functional limitations, which would give rise to a preference for the physically abled, non-obese informants when learning about novel physical activities. Because there is no association between general facts and one’s physical capability or body type, we anticipated that children would not show a strong preference for the physically abled, non-obese informants when learning about novel facts. Previous research has shown that by the preschool years, children do use the principle of familiarity or the cognitive division of labor as the basis for their judgments about who is more likely to possess specific knowledge (e.g., Lutz and Keil, 2002; Koenig and Jaswal, 2011; Ma and Woolley, 2013). The lack of a domain effect in Study 1, however, appears inconsistent with these hypotheses.

Why did children favor the physically abled, non-obese informants across both domains? One potential explanation is the halo effect, a cognitive bias in which people’s overall impression of a person influences their feelings and thoughts about specific traits of that person (Thorndike, 1920). For example, adults are more likely to ascribe positive qualities or traits to individuals that are physically attractive (e.g., Feingold, 1992). People also tend to perceive attractive individuals to be trustworthier than their unattractive counterparts (e.g., Wilson and Eckel, 2006). Developmental research has demonstrated that like adults, young children are also influenced by the halo effect in trait attribution. For example, 4- and 5-year-olds believe that a nice child would also be smarter and more athletic than a mean child, even though there is no causal link between one’s benevolence and intelligence or athletic ability (Cain et al., 1997). More relevant to the present study, it has been shown that 4- to 5-year-olds prefer to endorse the testimony of informants with more attractive faces (Bascandziev and Harris, 2014). In light of these findings, under the potential influence of the halo effect, the children in Study 1 might have had an overall positive stereotype of the physically abled, non-obese informants relative to the physically disabled or obese ones, which led them to attribute trustworthiness to the former group in the domains of both physical activities and facts.

The lack of a domain effect may also have something to do with a general negativity bias, which refers to people’s tendency to pay greater attention to, be influenced by, and have better memory recall of negative rather than positive information about the environment, which may increase the avoidance of negative events (Baumeister et al., 2001; Rozin and Royzman, 2001). This bias emerges early in development, probably late in the first year. For example, research on social referencing has demonstrated that by 12 months, infants show greater sensitivity to negative emotional cues such as fear and disgust rather than positive ones (see Vaish et al., 2008, for a review). As reviewed earlier, by the preschooler years, children have already developed negative stereotypes of physically disabled or obese others, as well as prejudice toward and discrimination against these individuals (e.g., Lerner and Korn, 1972; Cramer and Steinwert, 1998; Musher-Eizenman et al., 2004; Wei and Di Santo, 2012). Therefore, in the present study children might have attached overall negative valence to the physically disabled or obese informants and their testimony. Due to the general negativity bias, they might have avoided learning new knowledge from these informants.

In Study 2, we pitted physical characteristic against past reliability to further explore the robustness of children’s bias against the physically disabled or obese informants. When provided with cues that the physically disabled or obese informant was previously reliable whereas the physically abled, non-obese one was unreliable, children did not show a statistically significant preference for either informant. Past research has demonstrated that preschoolers prefer to endorse previously reliable rather than unreliable informants (e.g., Koenig et al., 2004; Harris and Koenig, 2006; Einav and Robinson, 2010; Liu et al., 2013). Moreover, it has been shown that past reliability seems a stronger indicator of informant trustworthiness for children than other cues (Jaswal and Neely, 2006; Corriveau et al., 2013), and that young children’s initial credulity toward the false testimony of an adult can be reversed by a single exposure to the adult being previously unreliable (Ma and Ganea, 2010). Given these findings, it is surprising that in Study 2 children did not show a significant preference for the physically disabled or obese informant who was previously reliable. Combined with the findings of Study 1, a tentative conclusion can be drawn that past reliability might undermine children’s bias against the physically disabled or obese informant, but cannot reverse it.

When considering the role of children’s prior exposure to people who are either physically disabled or obese, across both studies the children with and without relevant exposure responded similarly. However, it is worth noting that in Study 1, the children without relevant exposure preferred the physically abled, non-obese informants significantly above chance, whereas the children with relevant exposure made their choices randomly. It is possible that the children with relevant prior exposure might have had more positive overall impression of disabled or obese others than the children without relevant exposure, so they did not display a bias against these individuals as informants. However, caution should be taken when interpreting these results, as the sample size of the children with prior exposure to disabled others was relatively small (*n* = 11), so was the sample size of the children without prior exposure to obese others (*n* = 15). In addition, the difference in the responses of children with and without relevant exposure was not substantial, although statistically only the performance of the children without relevant exposure was above chance. In Study 2, both the children with and without relevant exposure responded at random, although we would expect that children with relevant exposure should show a preference for physically disabled or obese informants, based on their experiences with these populations as well as the evidence of their past reliability. Nevertheless, the present data provide some initial insight into the potential effect of prior exposure on children’s trust in physically disabled or obese informants, but the findings are still tentative.

Future examination is needed to address a few limitations in the current research. First, in both studies children were asked to make a choice between two informants who were placed in direct contrast with each other. It is possible that this forced choice would not reveal a complete picture of children’s trust in informants with or without physical disabilities or obesity, as there was no opportunity for a third option. To address this limitation, future work could adopt a paradigm that does not involve the direct contrast between two informants. For example, Ma and Ganea (2010) pitted young children’s firsthand observations against the false testimony of an adult to explore to what extent children would trust what they were told. It would be interesting to use this paradigm to find out whether children would place different levels of trust in the testimony of physically abled, non-obese informants versus that of physically disabled or obese ones, by contrasting their firsthand observations with misinformation from each of these two sources.

A second limitation concerns the images of the obese informants. In attempt to control for the potential influence of overall physical attractiveness, we edited the images of non-obese individuals to create the images of obese informants, which might appear distorted to some degree. Recall that each pair of informants were matched in terms of age, race, skin color, hair color, and outfits, and were rated by a team of viewers as equally confident in their claims and equally attractive before the photo editing took place. Future studies can use real images of individuals who are obese to be ecologically more valid. Nevertheless, we should mention that none of our participants and their parents reported that the images of the obese informants looked odd to them (in fact, some of them spontaneously described these informants as fat or chubby upon seeing their images).

Another limitation regards the data on children’s prior exposure. In both studies, the parent answered specific questions regarding if the child had been exposed to physically disabled or obese individuals (e.g., whether or not the child knows a person with disabilities or obesity, and if yes, who the person is and how often the child interacts with that person). However, some parents did not respond to any of these questions, resulting in the relatively small sample sizes as mentioned earlier. Some parents reported whether or not their children had prior exposure, but did not answer the other questions. Because of this, we lumped together the children who had a little exposure with the children who potentially had lots of exposure, which is not ideal as individual differences in the amount of prior exposure might result in different levels of selective learning. Thus, future research needs to use a more standard and comprehensive measure of children’s prior exposure to physically disabled or obese individuals, in order to better understand the effects of both the frequency and the nature of prior exposure on children’s selective learning from others.

Future research can take several other paths to provide more insight into how the physical capability or body type of informants influences children’s selective learning. The present data suggest that children might have a general bias against the physically disabled or obese informants, as they favored the physically abled, non-obese informants in the domains of both novel physical activities and novel facts (Study 1). It would be interesting to explore whether children would still show the same preference when learning about domains in which the physically disabled or obese informants are expected to have expertise (e.g., learning about how to use a wheelchair ramp). This would help elucidate whether children have a bias against physically disabled or obese informants in general, or whether there are circumstances in which children’s selective learning would be guided by epistemic prudence. Second, future work can look at the role of other forms of disabilities, such as facial disfigurements, leg amputations, and the use of crutches. Finally, it would be worth studying whether physically disabled or obese children also show less trust in informants who had the same functional limitations as themselves, given the research findings that even disabled or obese children themselves hold negative attitudes toward physically disabled or obese peers (Lerner and Korn, 1972; Richardson, 1983).

In conclusion, the present research will make new contributions to our understanding of children’s selective learning from others. Previous research has shown that preschool-aged children are able to selectively endorse information from others based on epistemic grounds such as past accuracy (e.g., Koenig et al., 2004). Our data demonstrate that when there is no indication of potential informants’ epistemic reliability, preschoolers may turn to visually salient physical differences in them, and make stereotypical judgments about whom to trust, under the potential influence of either a halo effect or a general negativity bias, or the combined force of both. Even when there is evidence that the physically disabled or obese informant might be more reliable than the physically abled, non-obese one, children still do not show a significant preference for the former. Thus, early-emerging social biases against physically disabled or obese individuals may be far-reaching and robust, and may foster selective learning from unfamiliar others based purely on non-epistemic grounds.

## Conflict of Interest Statement

The authors declare that the research was conducted in the absence of any commercial or financial relationships that could be construed as a potential conflict of interest.
